# Applying latent class assignments for accelerometry data to external populations: Data from the National Health and Nutrition Examination Survey 2003–2006

**DOI:** 10.1016/j.dib.2016.11.007

**Published:** 2016-11-09

**Authors:** Kelly R. Evenson, Fang Wen, Annie Green Howard, Amy H. Herring

**Affiliations:** a Department of Epidemiology, Gillings School of Global Public Health, University of North Carolina – Chapel Hill, Chapel Hill, North Carolina, United States; b Center for Health Promotion and Disease Prevention, University of North Carolina – Chapel Hill, Chapel Hill, North Carolina, United States; c Department of Biostatistics at the Gillings School of Global Public Health, Carolina Population Center, University of North Carolina – Chapel Hill, Chapel Hill, North Carolina, United States

**Keywords:** Epidemiology, Exercise, Latent class analysis, Physical activity, Mortality, Prevention, Sedentary behavior

## Abstract

Latent class analysis can identify unmeasured mutually exclusive categories (class membership) among participants for either observed categorical or continuous variables. More recently, latent class analysis has been applied to accelerometry to better understand the day-to-day patterns of physical activity and sedentary behavior. Typically, the class assignments are only relevant to the study for which they were derived and not made available for others to use. Using one-week accelerometry (ActiGraph #AM7164) data collected from the National Health and Nutrition Examination Survey during 2003–2006, latent classes of physical activity and sedentary behavior were derived separately for youths 6–17 years and adults >=18 years. The purpose of this article is to provide the latent class assignments developed on this source population (United States) available to others to apply to their studies using similarly collected accelerometry. This method will extend the usefulness of the latent class analysis and allow for comparisons across studies.

**Specifications Table**

**Table:** 

Subject area	Public health
More specific subject area	Accelerometry; physical activity; sedentary behavior
Type of data	Table, SAS files
How data was acquired	Public access data from the National Center for Health Statistics
Data format	Raw coded data
Experimental factors	Latent class assignments among youths and adults who participated in the National Health and Nutrition Examination Survey (NHANES) during 2003–2006 and wore an accelerometer for one week
Experimental features	Programs and an example dataset showing how to apply the latent class assignments to external populations
Data source location	United States (US)
Data accessibility	Data is available in the article.

**Value of the data**

• The latent class assignments can be applied to external populations of youths and adults that used similar accelerometry data collection. These classifications of individuals can then be used to describe patterns of accelerometer-assessed physical activity and sedentary behaviors among the study sample.
• By applying the latent class assignments, comparisons can be made between the external population and the assignments developed from a national data source (NHANES).
• The latent class assignments can also be applied without the intention of comparison. It will save the researcher time from having to develop and process the latent class models.

## 1 Data

Among the 2003–06 NHANES participants, 50 adults >=18 years wore an accelerometer and were purposefully selected to be in this example SAS dataset (example_adult_cpm.sas7bdat). Similarly, 50 youth 6–17 years wore an accelerometer and were purposefully selected to be in a second example SAS dataset (example_youth_cpm.sas7bdat). The example datasets are provided to demonstrate how the SAS macros can be implemented and used to apply the latent class assignments developed on this source population (US).

## 2 Experimental design, materials and methods

### 2.1 Data: Accelerometry

NHANES provides a cross-sectional assessment of the health of the US population. NHANES participants provided informed consent or parental assent, depending on age, before completing the questionnaires or measurements. The national data were deidentified and made publicly available through the US National Center for Health Statistics (http://www.cdc.gov/nchs/nhanes/). The latent class assignments were developed using latent class analysis, applied separately for youths [1] and adults [2] participating in NHANES.

From 2003–2006, participants age 6 years and older were asked to wear the ActiGraph accelerometer (#AM7164) on their hip for one week during waking hours and not during water-based activities. Beginning at midnight on the day following the clinic visit, the accelerometer recorded 1-minute epochs of data. In order to remove periods of non-wear, we applied a previously validated algorithm developed by Choi et al. [3] using SAS. Specifically, non-wear was defined by an interval of >=90 consecutive minutes of zero counts/minute, with allowance of up to 2 min of nonzero counts if no counts were detected during both the 30 min upstream and downstream from that interval. Any nonzero counts (except the allowed short intervals) were considered wear time. Counts in the non-wear period were set to missing.

For both youth and adults, we explored average physical activity intensity using average counts/minute. Next, we applied cutpoints to the data, separately for youth and adults, to obtain time spent in sedentary, light, moderate, and vigorous activity. The cutpoints applied to the data are summarized in Table 1.

### 2.2 Latent class analysis

After deriving the number of minutes/day for each day of the week spent in sedentary, light, moderate, and vigorous activity, we applied latent class analysis to develop natural groupings of participants who tended to accumulate their accelerometry behavior in a similar pattern. The application was performed using MPlus (version 7.11) [4], which allowed for the complex survey design of NHANES in conjunction with the modeling. Finite mixture models were used to describe the relationship between up to 7 adherent days of accelerometry and the categorical latent variable using a set of linear regression equations. The detail on the process to make the final selection of classes for each variable is described elsewhere [1], [2]. We assumed participants belonged to one of several mutually exclusive classes, but for which class membership was not known a priori. Each participant was assigned to one class for each measure with the highest posterior class membership probability.

Typically, latent class analysis is applied to a population and assignments are only relevant to the study for which they were derived. We developed a way for others to access and use the latent class assignments we previously developed on the national data. Thus, we have made available the programs to apply the latent class assignments to data collected similarly (Table 1 and Appendix A).

### 2.3 Study sample

We excluded participants who declined to participate in the accelerometry portion of the NHANES visit, or who returned the accelerometer after wearing it but it was found to be out of calibration or faulty upon return. We required >=3 adherent days with an adherent day indicated by >=8 h of accelerometer wear. For the youth, we also required that wear occur between 6:00 am to midnight. This same restriction was not applied to the adults since some participants, for example, may have been shift workers. This left a final sample size of 3998 youths and 7931 adults. Latent class analysis was applied to the overall sample, and among youths also stratified by gender, age (6–11, 12–14, 15–17 years), and whether or not they were attending school at the time of measurement.

### 2.4 Application

To apply the latent class assignments to accelerometry data similarly collected from the NHANES 2003–2006 accelerometry data, run the SAS code below to generate latent class variables and their corresponding posterior probabilities on average counts/min:

LIBNAME ANADT ‘<Full path to the example data folder>’;

%ADULT_LCA_OUT(INDT=ANADT.EXAMPLE_ADULT_DATA_CPM, UID=SEQN, NUMLC=6, PA=CNTMIN_, PATXT=CPM, OUTDT=LC_CNTMIN_OUT).

The SAS dataset (example_adult_data_cpm.sas7bdat) includes 50 participants that the user can plug in to test the macro before using.

In order to derive these variables on external data, we recommend that the age range is similar to those in our sample, that 3–7 consecutive days of accelerometry were collected, and that the accelerometer used is similar (ActiGraph).

## Figures and Tables

**Table 1:** Description of variables and corresponding programs and data derived among youths and adults participating in the accelerometry portion of NHANES 2003–2006.

	**Youth**	**Adults**
References	Development of latent classes [1]	Development of latent classes [2]; associations with correlates [5]; associations with mortality [6]
Age	6–17 years	18 years and older
Required wear time	>=3 or 7 days for >=8 h/day from 6:00am to midnight	>=3 or 7 days for >=8 h/day
Average intensity latent class variables	Average counts/minute/day	Average counts/minute/day
Cutpoint definitions for physical activity variables	Using [7]: light 100–2295 counts/min, moderate 2296–4011 counts/min, and vigorous >=4012 counts/min.	Using [8]: light 100–2019 counts/min, moderate 2020–5998 counts/min, and vigorous >=5999 counts/min. Using [9]: light 100–759 counts/min and MVPA >=760 counts/min.
Physical activity latent class variables	1. Percent of light activity out of total wearing time per day; 2. Percent of MVPA out of total wearing time per day; 3. Percent of vigorous activity out of total wearing time per day	1. Percent of MVPA out of total wearing time per day (using both sets of cutpoints [8], [9])
Cutpoint definitions for sedentary behavior variables	Using [7]: sedentary behavior <100 counts/min.	Using [10]: sedentary behavior <100 counts/min. Sedentary bouts were defined as >=30 min with at least 80% of the min falling below the sedentary threshold, allowing for <5 consecutive min above the threshold. The sedentary bout had to start and end with sedentary behavior.
Sedentary behavior latent class variables	1. Percent of sedentary behavior out of total wearing time per day	1. Percent of sedentary behavior out of total wearing time per day; 2. Percent of sedentary bouts out of total wearing time per day
Latent class variables available by strata	Yes for (1) gender; (2) age (6–11, 12–14, 15–17 years); and (3) in or out of school	no
		
Available through this Data in Brief:		
Documentation	Documentation_youth_LCA_macro	Documentation_adult_LCA_macro
SAS program	generate_LCA_Macros_NHANES_Youth.sas	generate_LCA_Macros_NHANES_Adults.sas
Example dataset in SAS to apply the SAS program to the macros	example_youth_cpm.sas7bdat	example_adult_cpm.sas7bdat
		
Statistics from MPlus output to derive latent class variables	stats_cpm.sas7bdat;	stats_cpm.sas7bdat;
	stats_lt.sas7bdat;	stats_mvcm.sas7bdat;
	stats_mvpa.sas7bdat;	stats_mvto.sas7bdat;
	stats_sd.sas7bdat;	stats_sd.sas7bdat;
	stats_vig.sas7bdat	stats_sdb.sas7bdat
	(plus additional statistics for in or out of school, boys or girls, and age 6–11, 12–14, and 15–17 years	

Abbreviations: MVPA, moderate to vigorous physical activity
