# Supplementary material for: Applying latent class assignments for accelerometry data to external populations: Data from the National Health and Nutrition Examination Survey 2003–2006
Source: Data Brief. 2016 Nov 9;9:926–30. doi: 10.1016/j.dib.2016.11.007 (PMC5118612; doi:10.1016/j.dib.2016.11.007)
Supplement: Supplementary file 1 — Supplementary material [file mmc3.zip › LCA_Package_Youth/Documentation_Youth_LCA_macro.pdf]

Youth Latent Classes Analysis Macro Documentation

Purpose:

This document describes the process of implementing a procedure to classify youth from an external study population into latent classes derived from national accelerometry data. These latent class assignments were derived from latent class analysis (LCA) using accelerometer data (ActiGraph AM7164) from youth 6-17 years old participating in the 2003-2006 National Health and Nutrition Examination Survey (NHANES), with details explained elsewhere (Evenson KR, Wen F, Hales D, Herring AH. National youth sedentary behavior and physical activity daily patterns using latent class analysis applied to accelerometry. Int J Behav Nutr Phys Act 2016;13(1):55). The macro assigns individuals to a latent class and provides the corresponding posterior probabilities based on their pattern of accelerometry over seven consecutive days.

The latent classes, and subsequently the macros, were derived for a number of different day-specific accelerometer measures including average counts per minute, percent of moderate to vigorous physical activity (MVPA) (>=2296 counts/minute), percent of light activity (100-2295 counts/minute), percent of vigorous activity (>=4012 counts/minute), and percent of sedentary behavior (<100 counts/minute). The measures were derived overall and by age (6-11, 12-14, 15-17), gender, and whether they were in or out of school at the time of the measurement. Due to the more flexible LCA options, the latent classes were generated using Mplus version 7.11, but the macro was developed for use in SAS. The cutpoint values were based on the following reference:
Evenson K, Catellier D, Gill K, Ondrak K, McMurray R. Calibration of two objective measures of physical activity for children. J Sport Sci. 2008;26(14):1557-65.

Prerequisites to apply the assignments to external study data:

- ✓ Require that the study data are on youth age 6-17 years old.
- ✓ Require that the study data contains up to 7 consecutive days of accelerometry from an ActiGraph accelerometer using the vertical axis only.
- ✓ Require that the accelerometer data are cleaned and summarized as follows:
    - Each participant has measures collected for 7 consecutive days on each target latent class measure, where accelerometry data are ordered by day of the week, with Sunday assigned as day 1, Monday as day 2, and so forth with Saturday as day 7.
    - Adherent data is defined as a participant who wore the accelerometer for at least 3 of 7 days for at least 8 hours per day. Non-adherent data is suggested to be excluded from the analysis. Any data on non-adherent days will be required to be set as missing (denoted as "." in SAS).

Data Dictionary and SAS Macro Application:

Step 1: Download the example dataset and required specificity statistics data listed below.

Summary of Files

Subfolder	File Name	Description
\PGM\	Generate_LCA_Macros_NHANES_Youth.sas	SAS program used to generate latent class variables.
\DATA\	example_youth_cpm.sas7bdat	Example SAS dataset to test the macro
\STATS\	stats_cpm.sas7bdat	Statistics extracted from Mplus LCA model on counts/minute
	stats_cpm_6to11.sas7bdat	Statistics extracted from Mplus LCA model on counts/minute on youth age 6-11 years old
	stats_cpm_12to14.sas7bdat	Statistics extracted from Mplus LCA model on counts/minute on youth age 12-14 years old
	stats_cpm_15to17.sas7bdat	Statistics extracted from Mplus LCA model on counts/min on youth age 15-17 years old
	stats_cpm_boys.sas7bdat	Statistics extracted from Mplus LCA model on counts/min on boys
	stats_cpm_girls.sas7bdat	Statistics extracted from Mplus LCA model on counts/min on girls
	stats_cpm_inschool.sas7bdat	Statistics extracted from Mplus LCA model on counts/min on youth in school
	stats_cpm_outschool.sas7bdat	Statistics extracted from Mplus LCA model on counts/min on youth out of school
	stats_lt.sas7bdat	Statistics extracted from Mplus LCA model on light activity
	stats_lt_6to11.sas7bdat	Statistics extracted from Mplus LCA model based on % light activity on youth age 6-11 years old
	stats_lt_12to14.sas7bdat	Statistics extracted from Mplus LCA model based on % light activity on youth age 12-14 years old
	stats_lt_15to17.sas7bdat	Statistics extracted from Mplus LCA model based on % light activity on youth age 15-17 years old
	stats_lt_boys.sas7bdat	Statistics extracted from Mplus LCA model based on % light activity on boys
	stats_lt_girls.sas7bdat	Statistics extracted from Mplus LCA model based on % light activity on girls
	stats_lt_inschool.sas7bdat	Statistics extracted from Mplus LCA model based on % light activity on youth in school
	stats_lt_outschool.sas7bdat	Statistics extracted from Mplus LCA model based on % light activity on youth out of school
	stats_mvpa.sas7bdat	Statistics extracted from Mplus LCA model on MVPA
	stats_mvpa_6to11.sas7bdat	Statistics extracted from Mplus LCA model based on % MVPA on youth age 6-11 years old
	stats_mvpa_12to14.sas7bdat	Statistics extracted from Mplus LCA model based on % MVPA on youth age 12-14 years old
	stats_mvpa_15to17.sas7bdat	Statistics extracted from Mplus LCA model based on % MVPA on youth age 15-17 years old
	stats_mvpa_boys.sas7bdat	Statistics extracted from Mplus LCA model based on % MVPA on boys
	stats_mvpa_girls.sas7bdat	Statistics extracted from Mplus LCA model based on % MVPA on girls

	stats_mvpa_inschool.sas7bdat	Statistics extracted from Mplus LCA model based on % MVPA on youth in school
	stats_mvpa_outschool.sas7bdat	Statistics extracted from Mplus LCA model based on % MVPA on youth out of school
	stats_vig.sas7bdat	Statistics extracted from Mplus LCA model on vigorous activity
	stats_vig_6to11.sas7bdat	Statistics extracted from Mplus LCA model on vigorous activity on youth age 6-11 years old
	stats_vig_12to14.sas7bdat	Statistics extracted from Mplus LCA model on vigorous activity on youth age 12-14 years old
	stats_vig_15to17.sas7bdat	Statistics extracted from Mplus LCA model on vigorous activity on youth age 15-17 years old
	stats_vig_boys.sas7bdat	Statistics extracted from Mplus LCA model on vigorous activity on boys
	stats_vig_girls.sas7bdat	Statistics extracted from Mplus LCA model on vigorous activity on girls
	stats_vig_inschool.sas7bdat	Statistics extracted from Mplus LCA model on vigorous activity on youth in school
	stats_vig_outschool.sas7bdat	Statistics extracted from Mplus LCA model on vigorous activity on youth out of school
	stats_sd.sas7bdat	Statistics extracted from Mplus LCA model based on % sedentary behavior
	stats_sd_6to11.sas7bdat	Statistics extracted from Mplus LCA model based on % sedentary behavior on youth age 6-11 years old
	stats_sd_12to14.sas7bdat	Statistics extracted from Mplus LCA model based on % sedentary behavior on youth age 12-14 years old
	stats_sd_15to17.sas7bdat	Statistics extracted from Mplus LCA model based on % sedentary behavior on youth age 15-17 years old
	stats_sd_boys.sas7bdat	Statistics extracted from Mplus LCA model based on % sedentary behavior on boys
	stats_sd_girls.sas7bdat	Statistics extracted from Mplus LCA model based on % sedentary behavior on girls
	stats_sd_inschool.sas7bdat	Statistics extracted from Mplus LCA model based on % sedentary behavior on youth in school
	stats_sd_outschool.sas7bdat	Statistics extracted from Mplus LCA model based on % sedentary behavior on youth out of school

Example Dataset
Among the 2003-06 NHANES participants, 50 youth 6-17 years old wore an accelerometer and were purposefully selected to be in this example dataset. The example dataset (example_youth_cpm.sas7bdat) is provided to demonstrate how the SAS macro can be implemented and used. A data dictionary for the dataset is provided below.

**Variable Name**	**Description**	**Values**
SEQN	Unique ID	Continuous
CNTMIN_1	Counts per minute on Sunday	Continuous
CNTMIN_2	Counts per minute on Monday	Continuous
CNTMIN_3	Counts per minute on Tuesday	Continuous
CNTMIN_4	Counts per minute on Wednesday	Continuous
CNTMIN_5	Counts per minute on Thursday	Continuous
CNTMIN_6	Counts per minute on Friday	Continuous
CNTMIN_7	Counts per minute on Saturday	Continuous

Step 2: Run the macro (Generate_LCA_Macros_NHANES_Youth.sas) on a specific accelerometer measure. The macro will be able to assign the most likely latent class to each individual as well as provide their corresponding associated posterior probabilities based on the following accelerometer measures:

- Average counts per minute - Age 6-17 years

  Example: in SAS, run the code below:
  %YOUTH_LCA_OUT(INDT=, UID=, NUMLC=4, PA= , PATXT=CPM, OUTDT=);
  Parameters input:
  INDT: (summarized) accelerometer data
  UID: unique ID of target study data
  NUMLC: number of latent classes assigned (Fixed, please do not change)
  PA: common pre-fix of 7-day accelerometer measures from the target study data
  PATXT: common character for names of latent class variable and its posterior probabilities
  (Fixed, please do not change)
  OUTDT: Output dataset with latent class variable and its posterior probabilities generated

  The following latent class variables in the table below will be generated in the dataset OUTDT accordingly.

**Variable Name**	**Description**	**Values**
**Latent classes on average counts per minute (CPM) - Overall**		
LC_CPM	Latent classes based on CPM	Categorical:<br>1 = Class 1<br>2 = Class 2<br>3 = Class 3<br>4 = Class 4
PP_CPM1	Posterior probability in latent class 1 -- based on CPM	Continuous
PP_CPM2	Posterior probability in latent class 2 -- based on CPM	Continuous
PP_CPM3	Posterior probability in latent class 3 -- based on CPM	Continuous
PP_CPM4	Posterior probability in latent class 4 -- based on CPM	Continuous

- Average counts per minute – Age 6 to 11 years

  Example: in SAS, please run the code below:
  %YOUTH_LCA_OUT(INDT=, UID=, NUMLC=3, PA= , PATXT=CPM_6to11, OUTDT=);
  Parameters input:
  INDT: (summarized) accelerometer data
  UID: unique ID of target study data
  NUMLC: number of latent classes assigned (Fixed, please do not change)
  PA: common pre-fix of 7-day accelerometer measures from the target study data
  PATXT: common character for names of latent class variable and its posterior probabilities
  (Fixed, please do not change)
  OUTDT: Output dataset with latent class variable and its posterior probabilities generated

  The following latent class variables in the table below will be generated in the dataset OUTDT accordingly.

Variable Name	Description	Values
**Latent classes on average counts per minute (CPM) – Age 6 to 11 years**		
LC_CPM _6to11	Latent classes based on CPM	Categorical:<br>1 = Class 1<br>2 = Class 2<br>3 = Class 3
PP_CPM _6to11_1	Posterior probability in latent class 1 -- based on CPM	Continuous
PP_CPM _6to11_2	Posterior probability in latent class 2 -- based on CPM	Continuous
PP_CPM _6to11_3	Posterior probability in latent class 3 -- based on CPM	Continuous

- Average counts per minute – Age 12-14 years

  Example: in SAS, please run the code below:
  %YOUTH_LCA_OUT(INDT=, UID=, NUMLC=3, PA= , PATXT=CPM_12to14, OUTDT=);
  Parameters input:
  INDT: (summarized) accelerometer data
  UID: unique ID of target study data
  NUMLC: number of latent classes assigned (Fixed, please do not change)
  PA: common pre-fix of 7-day accelerometer measures from the target study data
  PATXT: common character for names of latent class variable and its posterior probabilities
  (Fixed, please do not change)
  OUTDT: Output dataset with latent class variable and its posterior probabilities generated

  The following latent class variables in the table below will be generated in the dataset OUTDT accordingly.

Variable Name	Description	Values
**Latent classes on average counts per minute (CPM) – Age 12 to 14 years**		
LC3_CPM_12TO14	Latent classes based on CPM -- age 12 to 14 years	Categorical:<br>1 = Class 1<br>2 = Class 2<br>3 = Class 3
PP_CPM_12TO14_1	Posterior probability in latent class 1 -- based on CPM	Continuous

	(age 12-14)	
PP_CPM_12TO14_2	Posterior probability in latent class 2 -- based on CPM (age 12-14)	Continuous
PP_CPM_12TO14_3	Posterior probability in latent class 3 -- based on CPM (age 12-14)	Continuous

❖ <u>Average counts per minute – Age 15-17 years</u>

Example: in SAS, please run the code below:
%YOUTH_LCA_OUT(INDT=, UID=, NUMLC=3, PA= , PATXT=CPM_15to17, OUTDT=);
Parameters input:
INDT: (summarized) accelerometer data
UID: unique ID of target study data
NUMLC: number of latent classes assigned (Fixed, please do not change)
PA: common pre-fix of 7-day accelerometer measures from the target study data
PATXT: common character for names of latent class variable and its posterior probabilities
(Fixed, please do not change)
OUTDT: Output dataset with latent class variable and its posterior probabilities generated

The following latent class variables in the table below will be generated in the dataset OUTDT accordingly.

**Variable Name**	**Description**	**Values**
**Latent classes on average counts per minute (CPM) – Age 15 to 17 years**		
LC_CPM_15TO17	Latent classes based on CPM -- age 15 to 17 years	Categorical:<br>1 = Class 1<br>2 = Class 2<br>3 = Class 3
PP_CPM_15TO17_1	Posterior probability in latent class 1 -- based on CPM (age 15-17)	Continuous
PP_CPM_15TO17_2	Posterior probability in latent class 2 -- based on CPM (age 15-17)	Continuous
PP_CPM_15TO17_3	Posterior probability in latent class 3 -- based on CPM (age 15-17)	Continuous

❖ <u>Average counts per minute – Boys</u>

Example: in SAS, please run the code below:
%YOUTH_LCA_OUT(INDT=, UID=, NUMLC=4, PA= , PATXT=CPM_BOYS, OUTDT=);
Parameters input:
INDT: (summarized) accelerometer data
UID: unique ID of target study data
NUMLC: number of latent classes assigned (Fixed, please do not change)
PA: common pre-fix of 7-day accelerometer measures from the target study data
PATXT: common character for names of latent class variable and its posterior probabilities
(Fixed, please do not change)
OUTDT: Output dataset with latent class variable and its posterior probabilities generated

The following latent class variables in the table below will be generated in the dataset OUTDT accordingly.

Variable Name	Description	Values
**Latent classes on average counts per minute (CPM) - Boys**		
LC_CPM_BOYS	Latent classes based on CPM -- boys	Categorical:<br>1 = Class 1<br>2 = Class 2<br>3 = Class 3<br>4 = Class 4
PP_CPM_BOYS1	Posterior probability in latent class 1 -- based on CPM (boys)	Continuous
PP_CPM_BOYS2	Posterior probability in latent class 2 -- based on CPM (boys)	Continuous
PP_CPM_BOYS3	Posterior probability in latent class 3 -- based on CPM (boys)	Continuous
PP_CPM_BOYS4	Posterior probability in latent class 4 -- based on CPM (boys)	Continuous

❖ Average counts per minute – Girls

Example: in SAS, please run the code below:
%YOUTH_LCA_OUT(INDT=, UID=, NUMLC=3, PA= , PATXT=CPM_GIRLS, OUTDT=);
Parameters input:
INDT: (summarized) accelerometer data
UID: unique ID of target study data
NUMLC: number of latent classes assigned (Fixed, please do not change)
PA: common pre-fix of 7-day accelerometer measures from the target study data
PATXT: common character for names of latent class variable and its posterior probabilities (Fixed, please do not change)
OUTDT: Output dataset with latent class variable and its posterior probabilities generated

The following latent class variables in the table below will be generated in the dataset OUTDT accordingly.

Variable Name	Description	Values
**Latent classes on average counts per minute (CPM) - Girls**		
LC_CPM_GIRLS	Latent classes based on CPM -- girls	Categorical:<br>1 = Class 1<br>2 = Class 2<br>3 = Class 3
PP_CPM_GIRLS1	Posterior probability in latent class 1 -- based on CPM (girls)	Continuous
PP_CPM_GIRLS2	Posterior probability in latent class 2 -- based on CPM (girls)	Continuous
PP_CPM_GIRLS3	Posterior probability in latent class 3 -- based on CPM (girls)	Continuous

❖ <u>Average counts per minute – In school</u>

Example: in SAS, please run the code below:
%YOUTH_LCA_OUT(INDT=, UID=, NUMLC=4, PA= , PATXT=CPM_INSCH, OUTDT=);
Parameters input:
INDT: (summarized) accelerometer data
UID: unique ID of target study data
NUMLC: number of latent classes assigned (Fixed, please do not change)
PA: common pre-fix of 7-day accelerometer measures from the target study data
PATXT: common character for names of latent class variable and its posterior probabilities (Fixed, please do not change)
OUTDT: Output dataset with latent class variable and its posterior probabilities generated

The following latent class variables in the table below will be generated in the dataset OUTDT accordingly.

Variable Name	Description	Values
**Latent classes on average counts per minute (CPM) – in school**		
LC_CPM_INSCH	Latent classes based on CPM -- in school	Categorical:<br>1 = Class 1<br>2 = Class 2<br>3 = Class 3<br>4 = Class 4
PP_CPM_INSCH1	Posterior probability in latent class 1 -- based on CPM (in school)	Continuous
PP_CPM_INSCH2	Posterior probability in latent class 2 -- based on CPM (in school)	Continuous
PP_CPM_INSCH3	Posterior probability in latent class 3 -- based on CPM (in school)	Continuous
PP_CPM_INSCH4	Posterior probability in latent class 4 -- based on CPM (in school)	Continuous

❖ <u>Average counts per minute – Out of school</u>

Example: in SAS, please run the code below:
%YOUTH_LCA_OUT(INDT=, UID=, NUMLC=3, PA= , PATXT=CPM_OUTSCH, OUTDT=);
Parameters input:
INDT: (summarized) accelerometer data
UID: unique ID of target study data
NUMLC: number of latent classes assigned (Fixed, please do not change)
PA: common pre-fix of 7-day accelerometer measures from the target study data
PATXT: common character for names of latent class variable and its posterior probabilities (Fixed, please do not change)
OUTDT: Output dataset with latent class variable and its posterior probabilities generated

The following latent class variables in the table below will be generated in the dataset OUTDT accordingly.

Variable Name	Description	Values
**Latent classes on average counts per minute (CPM) – out of school**		
LC_CPM_OUTSCH	Latent classes based on CPM -- out of school	Categorical:<br>1 = Class 1<br>2 = Class 2<br>3 = Class 3
PP_CPM_OUTSCH1	Posterior probability in latent class 1 -- based on CPM (out of school)	Continuous
PP_CPM_OUTSCH2	Posterior probability in latent class 2 -- based on CPM (out of school)	Continuous
PP_CPM_OUTSCH3	Posterior probability in latent class 3 -- based on CPM (out of school)	Continuous

❖ <u>Percent of moderate to vigorous physical activity - Age 6-17 years (MVPA; >=2296 counts/minute) out of total wearing time</u>

Example: in SAS, please run the code below:
%YOUTH_LCA_OUT(INDT=, UID=, NUMLC=4, PA= , PATXT=MVPA, OUTDT=);
Parameters input:
INDT: (summarized) accelerometer data
UID: unique ID of target study data
NUMLC: number of latent classes assigned (Fixed, please do not change)
PA: common pre-fix of 7-day accelerometer measures from the target study data
PATXT: common character for names of latent class variable and its posterior probabilities (Fixed, please do not change)
OUTDT: Output dataset with latent class variable and its posterior probabilities generated

The following latent class variables in the table below will be generated in the dataset OUTDT accordingly.

Variable Name	Description	Values
**Latent classes on % MVPA (>=2296 counts/minute)**		
LC_MVPA	Latent classes based on %MVPA-- overall	Categorical:<br>1 = Class 1<br>2 = Class 2<br>3 = Class 3<br>4 = Class 4
PP_MVPA1	Posterior probability in latent class 1 -- on MVPA	Continuous
PP_MVPA2	Posterior probability in latent class 2 -- on MVPA	Continuous
PP_MVPA3	Posterior probability in latent class 3 -- on MVPA	Continuous
PP_MVPA4	Posterior probability in latent class 4 -- on MVPA	Continuous

❖ <u>Percent of moderate to vigorous physical activity – Age 6 to 11 years (MVPA; >=2296 counts/minute) out of total wearing time</u>

Example: in SAS, please run the code below:
%YOUTH_LCA_OUT(INDT=, UID=, NUMLC=3, PA= , PATXT=MVPA_6to11, OUTDT=);

Parameters input:
INDT: (summarized) accelerometer data
UID: unique ID of target study data
NUMLC: number of latent classes assigned (Fixed, please do not change)
PA: common pre-fix of 7-day accelerometer measures from the target study data
PATXT: common character for names of latent class variable and its posterior probabilities
(Fixed, please do not change)
OUTDT: Output dataset with latent class variable and its posterior probabilities generated

The following latent class variables in the table below will be generated in the dataset OUTDT accordingly.

Variable Name	Description	Values
**Latent classes on % MVPA (>=2296 counts/minute) – Age 6 to 11 years**		
LC_MVPA_6TO11	Latent classes based on %MVPA-- age 6 to 11 years	Categorical:<br>1 = Class 1<br>2 = Class 2<br>3 = Class 3
PP_MVPA_6to11_1	Posterior probability in latent class 1 -- based on %MVPA(age 6-11)	Continuous
PP_MVPA_6to11_2	Posterior probability in latent class 2 -- based on %MVPA(age 6-11)	Continuous
PP_MVPA_6to11_3	Posterior probability in latent class 3 -- based on %MVPA(age 6-11)	Continuous

❖ <u>Percent of moderate to vigorous physical activity – Age 12 to 14 years (MVPA; >=2296 counts/minute) out of total wearing time</u>

Example: in SAS, please run the code below:
%YOUTH_LCA_OUT(INDT=, UID=, NUMLC=3, PA= , PATXT=MVPA_12to14, OUTDT=);
Parameters input:
INDT: (summarized) accelerometer data
UID: unique ID of target study data
NUMLC: number of latent classes assigned (Fixed, please do not change)
PA: common pre-fix of 7-day accelerometer measures from the target study data
PATXT: common character for names of latent class variable and its posterior probabilities
(Fixed, please do not change)
OUTDT: Output dataset with latent class variable and its posterior probabilities generated

The following latent class variables in the table below will be generated in the dataset OUTDT accordingly.

Variable Name	Description	Values
**Latent classes on % MVPA (>=2296 counts/minute) – Age 12 to 14 years**		
LC_MVPA_12TO14	Latent classes based on %MVPA-- age 12 to 14 years	Categorical:<br>1 = Class 1<br>2 = Class 2<br>3 = Class 3
PP_MVPA_12TO14_1	Posterior probability in latent class 1 -- based	Continuous

	on %MVPA(age 12-14)	
PP_MVPA_12TO14_2	Posterior probability in latent class 2 -- based on %MVPA(age 12-14)	Continuous
PP_MVPA_12TO14_3	Posterior probability in latent class 3 -- based on %MVPA(age 12-14)	Continuous

❖ <u>Percent of moderate to vigorous physical activity – Age 15 to 17 years (MVPA; >=2296 counts/min) out of total wearing time</u>

Example: in SAS, please run the code below:
%YOUTH_LCA_OUT(INDT=, UID=, NUMLC=3, PA= , PATXT=MVPA_15to17, OUTDT=);
Parameters input:
INDT: (summarized) accelerometer data
UID: unique ID of target study data
NUMLC: number of latent classes assigned (Fixed, please do not change)
PA: common pre-fix of 7-day accelerometer measures from the target study data
PATXT: common character for names of latent class variable and its posterior probabilities (Fixed, please do not change)
OUTDT: Output dataset with latent class variable and its posterior probabilities generated

The following latent class variables in the table below will be generated in the dataset OUTDT accordingly.

**Variable Name**	**Description**	**Values**
**Latent classes on % MVPA (>=2296 counts/minute) – Age 15 to 17 years**		
LC_MVPA_15TO17	Latent classes based on %MVPA-- age 15 to 17 years	Categorical:<br>1 = Class 1<br>2 = Class 2<br>3 = Class 3
PP_MVPA_15TO17_1	Posterior probability in latent class 1 -- based on %MVPA(age 15-17)	Continuous
PP_MVPA_15TO17_2	Posterior probability in latent class 2 -- based on %MVPA(age 15-17)	Continuous
PP_MVPA_15TO17_3	Posterior probability in latent class 3 -- based on %MVPA(age 15-17)	Continuous

❖ <u>Percent of moderate to vigorous physical activity - Boys (MVPA; >=2296 counts/minute) out of total wearing time</u>

Example: in SAS, please run the code below:
%YOUTH_LCA_OUT(INDT=, UID=, NUMLC=4, PA= , PATXT=MVPA_BOYS, OUTDT=);
Parameters input:
INDT: (summarized) accelerometer data
UID: unique ID of target study data
NUMLC: number of latent classes assigned (Fixed, please do not change)
PA: common pre-fix of 7-day accelerometer measures from the target study data
PATXT: common character for names of latent class variable and its posterior probabilities (Fixed, please do not change)

OUTDT: Output dataset with latent class variable and its posterior probabilities generated

The following latent class variables in the table below will be generated in the dataset OUTDT accordingly.

Variable Name	Description	Values
**Latent classes on % MVPA (>=2296 counts/minute) - Boys**		
LC_MVPA_BOYS	Latent classes based on %MVPA-- boys	Categorical:<br>1 = Class 1<br>2 = Class 2<br>3 = Class 3<br>4 = Class 4
PP_MVPA_BOYS1	Posterior probability in latent class 1 -- based on %MVPA(boys)	Continuous
PP_MVPA_BOYS2	Posterior probability in latent class 2 -- based on %MVPA(boys)	Continuous
PP_MVPA_BOYS3	Posterior probability in latent class 3 -- based on %MVPA(boys)	Continuous
PP_MVPA_BOYS4	Posterior probability in latent class 4 -- based on %MVPA(boys)	Continuous

❖ <u>Percent of moderate to vigorous physical activity - Girls (MVPA; >=2296 counts/minute) out of total wearing time</u>

Example: in SAS, please run the code below:
%YOUTH_LCA_OUT(INDT=, UID=, NUMLC=3, PA= , PATXT=MVPA_GIRLS, OUTDT=);
Parameters input:
INDT: (summarized) accelerometer data
UID: unique ID of target study data
NUMLC: number of latent classes assigned (Fixed, please do not change)
PA: common pre-fix of 7-day accelerometer measures from the target study data
PATXT: common character for names of latent class variable and its posterior probabilities (Fixed, please do not change)
OUTDT: Output dataset with latent class variable and its posterior probabilities generated

The following latent class variables in the table below will be generated in the dataset OUTDT accordingly.

Variable Name	Description	Values
**Latent classes on % MVPA (>=2296 counts/minute) - Girls**		
LC_MVPA_GIRLS	Latent classes based on %MVPA-- girls	Categorical:<br>1 = Class 1<br>2 = Class 2<br>3 = Class 3
PP_MVPA_GIRLS1	Posterior probability in latent class 1 -- based on %MVPA(girls)	Continuous
PP_MVPA_GIRLS2	Posterior probability in latent class 2 -- based on %MVPA(girls)	Continuous

PP_MVPA_GIRLS3	Posterior probability in latent class 3 -- based on %MVPA(girls)	Continuous

❖ <u>Percent of moderate to vigorous physical activity – In school (MVPA; >=2296 counts/minute) out of total wearing time</u>

Example: in SAS, please run the code below:
%YOUTH_LCA_OUT(INDT=, UID=, NUMLC=3, PA= , PATXT=MVPA_INSCH, OUTDT=);
Parameters input:
INDT: (summarized) accelerometer data
UID: unique ID of target study data
NUMLC: number of latent classes assigned (Fixed, please do not change)
PA: common pre-fix of 7-day accelerometer measures from the target study data
PATXT: common character for names of latent class variable and its posterior probabilities
(Fixed, please do not change)
OUTDT: Output dataset with latent class variable and its posterior probabilities generated

The following latent class variables in the table below will be generated in the dataset OUTDT accordingly.

**Variable Name**	**Description**	**Values**
**Latent classes on % MVPA (>=2296 counts/minute) – In school**		
LC_MVPA_INSCH	Latent classes based on %MVPA-- in school	Categorical:<br>1 = Class 1<br>2 = Class 2<br>3 = Class 3<br>4 = Class 4
PP_MVPA_INSCH1	Posterior probability in latent class 1 -- based on %MVPA(in school)	Continuous
PP_MVPA_INSCH2	Posterior probability in latent class 2 -- based on %MVPA(in school)	Continuous
PP_MVPA_INSCH3	Posterior probability in latent class 3 -- based on %MVPA(in school)	Continuous

❖ <u>Percent of moderate to vigorous physical activity – Out of school (MVPA; >=2296 counts/min) out of total wearing time</u>

Example: in SAS, please run the code below:
%YOUTH_LCA_OUT(INDT=, UID=, NUMLC=3, PA= , PATXT=MVPA_INSCH, OUTDT=);
Parameters input:
INDT: (summarized) accelerometer data
UID: unique ID of target study data
NUMLC: number of latent classes assigned (Fixed, please do not change)
PA: common pre-fix of 7-day accelerometer measures from the target study data
PATXT: common character for names of latent class variable and its posterior probabilities
(Fixed, please do not change)
OUTDT: Output dataset with latent class variable and its posterior probabilities generated

The following latent class variables in the table below will be generated in the dataset OUTDT accordingly.

Variable Name	Description	Values
**Latent classes on % MVPA (>=2296 counts/minute) – Out of school**		
LC_MVPA_OUTSCH	Latent classes based on %MVPA-- out of school	Categorical:<br>1 = Class 1<br>2 = Class 2<br>3 = Class 3
PP_MVPA_OUTSCH1	Posterior probability in latent class 1 -- based on %MVPA(out of school)	Continuous
PP_MVPA_OUTSCH2	Posterior probability in latent class 2 -- based on %MVPA(out of school)	Continuous

- <u>Percent of light physical activity - Age 6-17 years (100-2295 counts/minute) out of total wearing time</u>

Example: in SAS, please run the code below:
%YOUTH_LCA_OUT(INDT=, UID=, NUMLC=4, PA= , PATXT=LT, OUTDT=);
Parameters input:
INDT: (summarized) accelerometer data
UID: unique ID of target study data
NUMLC: number of latent classes assigned (Fixed, please do not change)
PA: common pre-fix of 7-day accelerometer measures from the target study data
PATXT: common character for names of latent class variable and its posterior probabilities (Fixed, please do not change)
OUTDT: Output dataset with latent class variable and its posterior probabilities generated

The following latent class variables in the table below will be generated in the dataset OUTDT accordingly.

Variable Name	Description	Values
**Latent classes on % Light (100-2295 counts/minute)**		
LC_LT	Latent classes based on % Light activity -- overall	Categorical:<br>1 = Class 1<br>2 = Class 2<br>3 = Class 3<br>4 = Class 4
PP_LT1	Posterior probability in latent class 1 -- on Light activity	Continuous
PP_LT2	Posterior probability in latent class 2 -- on Light activity	Continuous
PP_LT3	Posterior probability in latent class 3 -- on Light activity	Continuous
PP_LT4	Posterior probability in latent class 4 -- on Light activity	Continuous

- <u>Percent of light physical activity – Age 6 to 11 years (100-2295 counts/minute) out of total wearing time</u>

Example: in SAS, please run the code below:
%YOUTH_LCA_OUT(INDT=, UID=, NUMLC=3, PA= , PATXT=LT_6to11, OUTDT=);
Parameters input:
INDT: (summarized) accelerometer data
UID: unique ID of target study data
NUMLC: number of latent classes assigned (Fixed, please do not change)
PA: common pre-fix of 7-day accelerometer measures from the target study data
PATXT: common character for names of latent class variable and its posterior probabilities (Fixed, please do not change)
OUTDT: Output dataset with latent class variable and its posterior probabilities generated

The following latent class variables in the table below will be generated in the dataset OUTDT accordingly.

**Variable Name**	**Description**	**Values**
**Latent classes on % Light (100-2295 counts/minute) – Age 6 to 11 years**		
LC_LT_6TO11	Latent classes based on % Light activity -- age 6 to 11 years	Categorical:<br>1 = Class 1<br>2 = Class 2<br>3 = Class 3
PP_LT_6to11_1	Posterior probability in latent class 1 -- based on % Light activity (age 6-11)	Continuous
PP_LT_6to11_2	Posterior probability in latent class 2 -- based on % Light activity (age 6-11)	Continuous
PP_LT_6to11_3	Posterior probability in latent class 3 -- based on % Light activity (age 6-11)	Continuous

- <u>Percent of light physical activity – Age 12 to 14 years (100-2295 counts/minute) out of total wearing time</u>

Example: in SAS, please run the code below:
%YOUTH_LCA_OUT(INDT=, UID=, NUMLC=3, PA= , PATXT=LT_12to14, OUTDT=);
Parameters input:
INDT: (summarized) accelerometer data
UID: unique ID of target study data
NUMLC: number of latent classes assigned (Fixed, please do not change)
PA: common pre-fix of 7-day accelerometer measures from the target study data
PATXT: common character for names of latent class variable and its posterior probabilities (Fixed, please do not change)
OUTDT: Output dataset with latent class variable and its posterior probabilities generated

The following latent class variables in the table below will be generated in the dataset OUTDT accordingly.

**Variable Name**	**Description**	**Values**
**Latent classes on % Light (100-2295 counts/minute) – Age 12 to 14 years**		

LC_LT_12TO14	Latent classes based on % Light activity -- age 12 to 14 years	Categorical:<br>1 = Class 1<br>2 = Class 2<br>3 = Class 3
PP_LT_12TO14_1	Posterior probability in latent class 1 -- based on % Light activity (age 12-14)	Continuous
PP_LT_12TO14_2	Posterior probability in latent class 2 -- based on % Light activity (age 12-14)	Continuous
PP_LT_12TO14_3	Posterior probability in latent class 3 -- based on % Light activity (age 12-14)	Continuous

- Percent of light physical activity – Age 15 to 17 years (100-2295 counts/minute) out of total wearing time

Example: in SAS, please run the code below:
%YOUTH_LCA_OUT(INDT=, UID=, NUMLC=3, PA= , PATXT=LT_15to17, OUTDT=);
Parameters input:
INDT: (summarized) accelerometer data
UID: unique ID of target study data
NUMLC: number of latent classes assigned (Fixed, please do not change)
PA: common pre-fix of 7-day accelerometer measures from the target study data
PATXT: common character for names of latent class variable and its posterior probabilities (Fixed, please do not change)
OUTDT: Output dataset with latent class variable and its posterior probabilities generated

The following latent class variables in the table below will be generated in the dataset OUTDT accordingly.

Variable Name	Description	Values
**Latent classes on % Light (100-2295 counts/minute) – Age 15 to 17 years**		
LC_LT_15TO17	Latent classes based on % Light activity -- age 15 to 17 years	Categorical:<br>1 = Class 1<br>2 = Class 2<br>3 = Class 3
PP_LT_15TO17_1	Posterior probability in latent class 1 -- based on % Light activity (age 15-17)	Continuous
PP_LT_15TO17_2	Posterior probability in latent class 2 -- based on % Light activity (age 15-17)	Continuous
PP_LT_15TO17_3	Posterior probability in latent class 3 -- based on % Light activity (age 15-17)	Continuous

- Percent of light physical activity – Boys (100-2295 counts/minute) out of total wearing time

Example: in SAS, please run the code below:
%YOUTH_LCA_OUT(INDT=, UID=, NUMLC=4, PA= , PATXT=LT_BOYS, OUTDT=);
Parameters input:
INDT: (summarized) accelerometer data

UID: unique ID of target study data
NUMLC: number of latent classes assigned (Fixed, please do not change)
PA: common pre-fix of 7-day accelerometer measures from the target study data
PATXT: common character for names of latent class variable and its posterior probabilities (Fixed, please do not change)
OUTDT: Output dataset with latent class variable and its posterior probabilities generated

The following latent class variables in the table below will be generated in the dataset OUTDT accordingly.

**Variable Name**	**Description**	**Values**
**Latent classes on % Light (100-2295 counts/minute) - Boys**		
LC_LT_BOYS	Latent classes based on % Light activity -- boys	Categorical:<br>1 = Class 1<br>2 = Class 2<br>3 = Class 3<br>4 = Class 4
PP_LT_BOYS1	Posterior probability in latent class 1 -- based on % Light activity (boys)	Continuous
PP_LT_BOYS2	Posterior probability in latent class 2 -- based on % Light activity (boys)	Continuous
PP_LT_BOYS3	Posterior probability in latent class 3 -- based on % Light activity (boys)	Continuous
PP_LT_BOYS4	Posterior probability in latent class 4 -- based on % Light activity (boys)	Continuous

- <u>Percent of light physical activity – Girls (100-2295 counts/minute) out of total wearing time</u>

Example: in SAS, please run the code below:
%YOUTH_LCA_OUT(INDT=, UID=, NUMLC=4, PA= , PATXT=LT_GIRLS, OUTDT=);
Parameters input:
INDT: (summarized) accelerometer data
UID: unique ID of target study data
NUMLC: number of latent classes assigned (Fixed, please do not change)
PA: common pre-fix of 7-day accelerometer measures from the target study data
PATXT: common character for names of latent class variable and its posterior probabilities (Fixed, please do not change)
OUTDT: Output dataset with latent class variable and its posterior probabilities generated

The following latent class variables in the table below will be generated in the dataset OUTDT accordingly.

**Variable Name**	**Description**	**Values**
**Latent classes on % Light (100-2295 counts/minute) - Girls**		

LC_LT_GIRLS	Latent classes based on % Light activity -- girls	Categorical:<br>1 = Class 1<br>2 = Class 2<br>3 = Class 3<br>4 = Class 4
PP_LT_GIRLS1	Posterior probability in latent class 1 -- based on % Light activity (girls)	Continuous
PP_LT_GIRLS2	Posterior probability in latent class 2 -- based on % Light activity (girls)	Continuous
PP_LT_GIRLS3	Posterior probability in latent class 3 -- based on % Light activity (girls)	Continuous
PP_LT_GIRLS4	Posterior probability in latent class 4 -- based on % Light activity (girls)	Continuous

❖ Percent of light physical activity – In school (100-2295 counts/minute) out of total wearing time

Example: in SAS, please run the code below:
%YOUTH_LCA_OUT(INDT=, UID=, NUMLC=4, PA= , PATXT=LT_INSCH, OUTDT=);
Parameters input:
INDT: (summarized) accelerometer data
UID: unique ID of target study data
NUMLC: number of latent classes assigned (Fixed, please do not change)
PA: common pre-fix of 7-day accelerometer measures from the target study data
PATXT: common character for names of latent class variable and its posterior probabilities (Fixed, please do not change)
OUTDT: Output dataset with latent class variable and its posterior probabilities generated

The following latent class variables in the table below will be generated in the dataset OUTDT accordingly.

Variable Name	Description	Values
**Latent classes on % Light (100-2295 counts/minute) – In school**		
LC_LT_INSCH	Latent classes based on % Light activity -- in school	Categorical:<br>1 = Class 1<br>2 = Class 2<br>3 = Class 3<br>4 = Class 4
PP_LT_INSCH1	Posterior probability in latent class 1 -- based on % Light activity (in school)	Continuous
PP_LT_INSCH2	Posterior probability in latent class 2 -- based on % Light activity (in school)	Continuous
PP_LT_INSCH3	Posterior probability in latent class 3 -- based on % Light activity (in school)	Continuous
PP_LT_INSCH4	Posterior probability in latent class 4 -- based on % Light activity (in school)	Continuous

❖ Percent of light physical activity – Out of school (100-2295 counts/minute) out of total wearing time

Example: in SAS, please run the code below:
%YOUTH_LCA_OUT(INDT=, UID=, NUMLC=3, PA= , PATXT=LT_GIRLS, OUTDT=);
Parameters input:
INDT: (summarized) accelerometer data
UID: unique ID of target study data
NUMLC: number of latent classes assigned (Fixed, please do not change)
PA: common pre-fix of 7-day accelerometer measures from the target study data
PATXT: common character for names of latent class variable and its posterior probabilities (Fixed, please do not change)
OUTDT: Output dataset with latent class variable and its posterior probabilities generated

The following latent class variables in the table below will be generated in the dataset OUTDT accordingly.

**Variable Name**	**Description**	**Values**
**Latent classes on % Light (100-2295 counts/minute) – Out of school**		
LC_LT_OUTSCH	Latent classes based on % Light activity -- out of school	Categorical:<br>1 = Class 1<br>2 = Class 2<br>3 = Class 3
PP_LT_OUTSCH1	Posterior probability in latent class 1 -- based on % Light activity (out of school)	Continuous
PP_LT_OUTSCH2	Posterior probability in latent class 2 -- based on % Light activity (out of school)	Continuous
PP_LT_OUTSCH3	Posterior probability in latent class 3 -- based on % Light activity (out of school)	Continuous

- Percent of sedentary behavior - Age 6-17 years (<100 counts/minute) out of total wearing time

Example: in SAS, please run the code below:
%YOUTH_LCA_OUT(INDT=, UID=, NUMLC=4, PA= , PATXT=SD, OUTDT=);
Parameters input:
INDT: (summarized) accelerometer data
UID: unique ID of target study data
NUMLC: number of latent classes assigned (Fixed, please do not change)
PA: common pre-fix of 7-day accelerometer measures from the target study data
PATXT: common character for names of latent class variable and its posterior probabilities (Fixed, please do not change)
OUTDT: Output dataset with latent class variable and its posterior probabilities generated

The following latent class variables in the table below will be generated in the dataset OUTDT accordingly.

**Variable Name**	**Description**	**Values**
**Latent classes on % Sedentary behavior**		

LC_SD	Latent classes based on % Sedentary behavior -- overall	Categorical:<br>1 = Class 1<br>2 = Class 2<br>3 = Class 3<br>4 = Class 4
PP_SD1	Posterior probability in latent class 1 -- based on % Sedentary behavior	Continuous
PP_SD2	Posterior probability in latent class 2 -- based on % Sedentary behavior	Continuous
PP_SD3	Posterior probability in latent class 3 -- based on % Sedentary behavior	Continuous
PP_SD4	Posterior probability in latent class 4 -- based on % Sedentary behavior	Continuous

❖ <u>Percent of sedentary behavior – Age 6 to 11 years (<100 counts/minute) out of total wearing time</u>

Example: in SAS, please run the code below:
%YOUTH_LCA_OUT(INDT=, UID=, NUMLC=4, PA= , PATXT=SD_6to11, OUTDT=);
Parameters input:
INDT: (summarized) accelerometer data
UID: unique ID of target study data
NUMLC: number of latent classes assigned (Fixed, please do not change)
PA: common pre-fix of 7-day accelerometer measures from the target study data
PATXT: common character for names of latent class variable and its posterior probabilities (Fixed, please do not change)
OUTDT: Output dataset with latent class variable and its posterior probabilities generated

The following latent class variables in the table below will be generated in the dataset OUTDT accordingly.

**Variable Name**	**Description**	**Values**
**Latent classes on % Sedentary behavior – Age 6 to 11 years**		
LC_SD_6TO11	Latent classes based on % Sedentary behavior -- age 6 to 11 years	Categorical:<br>1 = Class 1<br>2 = Class 2<br>3 = Class 3<br>4 = Class 4
PP_SD_6to11_1	Posterior probability in latent class 1 -- based on % Sedentary behavior (age 6-11)	Continuous
PP_SD_6to11_2	Posterior probability in latent class 2 -- based on % Sedentary behavior (age 6-11)	Continuous
PP_SD_6to11_3	Posterior probability in latent class 3 -- based on % Sedentary behavior (age 6-11)	Continuous
PP_SD_6to11_4	Posterior probability in latent class 4 -- based on % Sedentary behavior (age 6-11)	Continuous

❖ <u>Percent of sedentary behavior – Age 12 to 14 years (<100 counts/minute) out of total wearing time</u>

Example: in SAS, please run the code below:
%YOUTH_LCA_OUT(INDT=, UID=, NUMLC=4, PA= , PATXT=SD_12to14, OUTDT=);
Parameters input:
INDT: (summarized) accelerometer data
UID: unique ID of target study data
NUMLC: number of latent classes assigned (Fixed, please do not change)
PA: common pre-fix of 7-day accelerometer measures from the target study data
PATXT: common character for names of latent class variable and its posterior probabilities (Fixed, please do not change)
OUTDT: Output dataset with latent class variable and its posterior probabilities generated

The following latent class variables in the table below will be generated in the dataset OUTDT accordingly.

Variable Name	Description	Values
**Latent classes on % Sedentary behavior – Age 12 to 14 years**		
LC_SD_12TO14	Latent classes based on % Sedentary behavior -- age 12 to 14 years	Categorical:<br>1 = Class 1<br>2 = Class 2<br>3 = Class 3<br>4 = Class 4
PP_SD_12TO14_1	Posterior probability in latent class 1 -- based on % Sedentary behavior (age 12-14)	Continuous
PP_SD_12TO14_2	Posterior probability in latent class 2 -- based on % Sedentary behavior (age 12-14)	Continuous
PP_SD_12TO14_3	Posterior probability in latent class 3 -- based on % Sedentary behavior (age 12-14)	Continuous
PP_SD_12TO14_4	Posterior probability in latent class 4 -- based on % Sedentary behavior (age 12-14)	Continuous

❖ <u>Percent of sedentary behavior – Age 15 to 17 years (<100 counts/minute) out of total wearing time</u>

Example: in SAS, please run the code below:
%YOUTH_LCA_OUT(INDT=, UID=, NUMLC=4, PA= , PATXT=SD_15to17, OUTDT=);
Parameters input:
INDT: (summarized) accelerometer data
UID: unique ID of target study data
NUMLC: number of latent classes assigned (Fixed, please do not change)
PA: common pre-fix of 7-day accelerometer measures from the target study data
PATXT: common character for names of latent class variable and its posterior probabilities (Fixed, please do not change)
OUTDT: Output dataset with latent class variable and its posterior probabilities generated

The following latent class variables in the table below will be generated in the dataset OUTDT accordingly.

Variable Name	Description	Values
**Latent classes on % Sedentary behavior – Age 15 to 17 years**		

LC_SD_15TO17	Latent classes based on % Sedentary behavior -- age 15 to 17 years	Categorical:<br>1 = Class 1<br>2 = Class 2<br>3 = Class 3<br>4 = Class 4
PP_SD_15TO17_1	Posterior probability in latent class 1 -- based on % Sedentary behavior (age 15-17)	Continuous
PP_SD_15TO17_2	Posterior probability in latent class 2 -- based on % Sedentary behavior (age 15-17)	Continuous
PP_SD_15TO17_3	Posterior probability in latent class 3 -- based on % Sedentary behavior (age 15-17)	Continuous
PP_SD_15TO17_4	Posterior probability in latent class 4 -- based on % Sedentary behavior (age 15-17)	Continuous

❖ <u>Percent of sedentary behavior – Boys (<100 counts/minute) out of total wearing time</u>

Example: in SAS, please run the code below:
%YOUTH_LCA_OUT(INDT=, UID=, NUMLC=4, PA= , PATXT=SD_BOYS, OUTDT=);
Parameters input:
INDT: (summarized) accelerometer data
UID: unique ID of target study data
NUMLC: number of latent classes assigned (Fixed, please do not change)
PA: common pre-fix of 7-day accelerometer measures from the target study data
PATXT: common character for names of latent class variable and its posterior probabilities (Fixed, please do not change)
OUTDT: Output dataset with latent class variable and its posterior probabilities generated

The following latent class variables in the table below will be generated in the dataset OUTDT accordingly.

Variable Name	Description	Values
**Latent classes on % Sedentary behavior – Boys**		
LC_SD_BOYS	Latent classes based on % Sedentary behavior -- boys	Categorical:<br>1 = Class 1<br>2 = Class 2<br>3 = Class 3<br>4 = Class 4
PP_SD_BOYS1	Posterior probability in latent class 1 -- based on % Sedentary behavior (boys)	Continuous
PP_SD_BOYS2	Posterior probability in latent class 2 -- based on % Sedentary behavior (boys)	Continuous
PP_SD_BOYS3	Posterior probability in latent class 3 -- based on % Sedentary behavior (boys)	Continuous
PP_SD_BOYS4	Posterior probability in latent class 4 -- based on % Sedentary behavior (boys)	Continuous

❖ Percent of sedentary behavior – Girls (<100 counts/minute) out of total wearing time

Example: in SAS, please run the code below:
%YOUTH_LCA_OUT(INDT=, UID=, NUMLC=4, PA= , PATXT=SD_GIRLS, OUTDT=);
Parameters input:
INDT: (summarized) accelerometer data
UID: unique ID of target study data
NUMLC: number of latent classes assigned (Fixed, please do not change)
PA: common pre-fix of 7-day accelerometer measures from the target study data
PATXT: common character for names of latent class variable and its posterior probabilities (Fixed, please do not change)
OUTDT: Output dataset with latent class variable and its posterior probabilities generated

The following latent class variables in the table below will be generated in the dataset OUTDT accordingly.

Variable Name	Description	Values
**Latent classes on % Sedentary behavior – Girls**		
LC_SD_GIRLS	Latent classes based on % Sedentary behavior -- girls	Categorical:<br>1 = Class 1<br>2 = Class 2<br>3 = Class 3<br>4 = Class 4
PP_SD_GIRLS1	Posterior probability in latent class 1 -- based on % Sedentary behavior (girls)	Continuous
PP_SD_GIRLS2	Posterior probability in latent class 2 -- based on % Sedentary behavior (girls)	Continuous
PP_SD_GIRLS3	Posterior probability in latent class 3 -- based on % Sedentary behavior (girls)	Continuous
PP_SD_GIRLS4	Posterior probability in latent class 4 -- based on % Sedentary behavior (girls)	Continuous

❖ Percent of sedentary behavior – In school (<100 counts/minute) out of total wearing time

Example: in SAS, please run the code below:
%YOUTH_LCA_OUT(INDT=, UID=, NUMLC=4, PA= , PATXT=SD_INSCH, OUTDT=);
Parameters input:
INDT: (summarized) accelerometer data
UID: unique ID of target study data
NUMLC: number of latent classes assigned (Fixed, please do not change)
PA: common pre-fix of 7-day accelerometer measures from the target study data
PATXT: common character for names of latent class variable and its posterior probabilities (Fixed, please do not change)
OUTDT: Output dataset with latent class variable and its posterior probabilities generated

The following latent class variables in the table below will be generated in the dataset OUTDT accordingly.

Variable Name	Description	Values
**Latent classes on % Sedentary behavior – In school**		
LC_SD_INSCH	Latent classes based on % Sedentary behavior -- in school	Categorical:<br>1 = Class 1<br>2 = Class 2<br>3 = Class 3<br>4 = Class 4
PP_SD_INSCH1	Posterior probability in latent class 1 -- based on % Sedentary behavior (in school)	Continuous
PP_SD_INSCH2	Posterior probability in latent class 2 -- based on % Sedentary behavior (in school)	Continuous
PP_SD_INSCH3	Posterior probability in latent class 3 -- based on % Sedentary behavior (in school)	Continuous
PP_SD_INSCH4	Posterior probability in latent class 4 -- based on % Sedentary behavior (in school)	Continuous

- <u>Percent of sedentary behavior – Out of school (<100 counts/minute) out of total wearing time</u>

Example: in SAS, please run the code below:
%YOUTH_LCA_OUT(INDT=, UID=, NUMLC=3, PA= , PATXT=SD_GIRLS, OUTDT=);
Parameters input:
INDT: (summarized) accelerometer data
UID: unique ID of target study data
NUMLC: number of latent classes assigned (Fixed, please do not change)
PA: common pre-fix of 7-day accelerometer measures from the target study data
PATXT: common character for names of latent class variable and its posterior probabilities (Fixed, please do not change)
OUTDT: Output dataset with latent class variable and its posterior probabilities generated

The following latent class variables in the table below will be generated in the dataset OUTDT accordingly.

Variable Name	Description	Values
**Latent classes on % Sedentary behavior – Out of school**		
LC_SD_OUTSCH	Latent classes based on % Sedentary behavior -- out of school	Categorical:<br>1 = Class 1<br>2 = Class 2<br>3 = Class 3
PP_SD_OUTSCH1	Posterior probability in latent class 1 -- based on % Sedentary behavior (out of school)	Continuous
PP_SD_OUTSCH2	Posterior probability in latent class 2 -- based on % Sedentary behavior (out of school)	Continuous
PP_SD_OUTSCH3	Posterior probability in latent class 3 -- based on % Sedentary behavior (out of school)	Continuous

❖ <u>Percent of vigorous activity - Age 6-17 years (>=4012 counts/minute) out of total wearing time</u>

Example: in SAS, please run the code below:
%YOUTH_LCA_OUT(INDT=, UID=, NUMLC=3, PA= , PATXT=VIG, OUTDT=);
Parameters input:
INDT: (summarized) accelerometer data
UID: unique ID of target study data
NUMLC: number of latent classes assigned (Fixed, please do not change)
PA: common pre-fix of 7-day accelerometer measures from the target study data
PATXT: common character for names of latent class variable and its posterior probabilities (Fixed, please do not change)
OUTDT: Output dataset with latent class variable and its posterior probabilities generated

The following latent class variables in the table below will be generated in the dataset OUTDT accordingly.

Variable Name	Description	Values
**Latent classes on % Vigorous activity**		
LC_VIG	Latent classes based on % Vigorous activity -- overall	Categorical:<br>1 = Class 1<br>2 = Class 2<br>3 = Class 3
PP_VIG1	Posterior probability in latent class 1 -- based on % Vigorous activity	Continuous
PP_VIG2	Posterior probability in latent class 2 -- based on % Vigorous activity	Continuous
PP_VIG3	Posterior probability in latent class 3 -- based on % Vigorous activity	Continuous

❖ <u>Percent of vigorous activity – Age 6 to 11 years (>=4012 counts/minute) out of total wearing time</u>

Example: in SAS, please run the code below:
%YOUTH_LCA_OUT(INDT=, UID=, NUMLC=3, PA= , PATXT=VIG_6to11, OUTDT=);
Parameters input:
INDT: (summarized) accelerometer data
UID: unique ID of target study data
NUMLC: number of latent classes assigned (Fixed, please do not change)
PA: common pre-fix of 7-day accelerometer measures from the target study data
PATXT: common character for names of latent class variable and its posterior probabilities (Fixed, please do not change)
OUTDT: Output dataset with latent class variable and its posterior probabilities generated

The following latent class variables in the table below will be generated in the dataset OUTDT accordingly.

Variable Name	Description	Values
**Latent classes on % Vigorous activity – Age 6 to 11 years**		
LC_VIG_6TO11	Latent classes based on % Vigorous activity -- age 6 to 11 years	Categorical:<br>1 = Class 1<br>2 = Class 2<br>3 = Class 3
PP_VIG_6to11_1	Posterior probability in latent class 1 -- based on % Vigorous activity (age 6-11)	Continuous
PP_VIG_6to11_2	Posterior probability in latent class 2 -- based on % Vigorous activity (age 6-11)	Continuous
PP_VIG_6to11_3	Posterior probability in latent class 3 -- based on % Vigorous activity (age 6-11)	Continuous

- Percent of vigorous activity – Age 12 to 14 years (>=4012 counts/minute) out of total wearing time

Example: in SAS, please run the code below:
%YOUTH_LCA_OUT(INDT=, UID=, NUMLC=2, PA= , PATXT=VIG_12to14, OUTDT=);
Parameters input:
INDT: (summarized) accelerometer data
UID: unique ID of target study data
NUMLC: number of latent classes assigned (Fixed, please do not change)
PA: common pre-fix of 7-day accelerometer measures from the target study data
PATXT: common character for names of latent class variable and its posterior probabilities (Fixed, please do not change)
OUTDT: Output dataset with latent class variable and its posterior probabilities generated

The following latent class variables in the table below will be generated in the dataset OUTDT accordingly.

Variable Name	Description	Values
**Latent classes on % Vigorous activity – Age 12 to 14 years**		
LC_VIG_12TO14	Latent classes based on % Vigorous activity -- age 12 to 14 years	Categorical:<br>1 = Class 1<br>2 = Class 2
PP_VIG_12TO14_1	Posterior probability in latent class 1 -- based on % Vigorous activity (age 12-14)	Continuous
PP_VIG_12TO14_2	Posterior probability in latent class 2 -- based on % Vigorous activity (age 12-14)	Continuous

- Percent of vigorous activity – Age 15 to 17 years (>=4012 counts/minute) out of total wearing time

Example: in SAS, please run the code below:
%YOUTH_LCA_OUT(INDT=, UID=, NUMLC=3, PA= , PATXT=VIG_15to17, OUTDT=);
Parameters input:
INDT: (summarized) accelerometer data
UID: unique ID of target study data

NUMLC: number of latent classes assigned (Fixed, please do not change)
PA: common pre-fix of 7-day accelerometer measures from the target study data
PATXT: common character for names of latent class variable and its posterior probabilities
(Fixed, please do not change)
OUTDT: Output dataset with latent class variable and its posterior probabilities generated

The following latent class variables in the table below will be generated in the dataset OUTDT accordingly.

Variable Name	Description	Values
**Latent classes on % Vigorous activity – Age 15 to 17 years**		
LC_VIG_15TO17	Latent classes based on % Vigorous activity -- age 15 to 17 years	Categorical:<br>1 = Class 1<br>2 = Class 2<br>3 = Class 3
PP_VIG_15TO17_1	Posterior probability in latent class 1 -- based on % Vigorous activity (age 15-17)	Continuous
PP_VIG_15TO17_2	Posterior probability in latent class 2 -- based on % Vigorous activity (age 15-17)	Continuous
PP_VIG_15TO17_3	Posterior probability in latent class 3 -- based on % Vigorous activity (age 15-17)	Continuous

❖ <u>Percent of vigorous activity – Boys (>=4012 counts/minute) out of total wearing time</u>

Example: in SAS, please run the code below:
%YOUTH_LCA_OUT(INDT=, UID=, NUMLC=3, PA= , PATXT=VIG_BOYS, OUTDT=);
Parameters input:
INDT: (summarized) accelerometer data
UID: unique ID of target study data
NUMLC: number of latent classes assigned (Fixed, please do not change)
PA: common pre-fix of 7-day accelerometer measures from the target study data
PATXT: common character for names of latent class variable and its posterior probabilities
(Fixed, please do not change)
OUTDT: Output dataset with latent class variable and its posterior probabilities generated

The following latent class variables in the table below will be generated in the dataset OUTDT accordingly.

Variable Name	Description	Values
**Latent classes on % Vigorous activity – Boys**		
LC_VIG_BOYS	Latent classes based on % Vigorous activity -- boys	Categorical:<br>1 = Class 1<br>2 = Class 2<br>3 = Class 3
PP_VIG_BOYS1	Posterior probability in latent class 1 -- based on % Vigorous activity (boys)	Continuous
PP_VIG_BOYS2	Posterior probability in latent class 2 -- based on % Vigorous activity (boys)	Continuous

PP_VIG_BOYS3	Posterior probability in latent class 3 -- based on % Vigorous activity (boys)	Continuous

❖ <u>Percent of vigorous activity – Girls (>=4012 counts/minute) out of total wearing time</u>

Example: in SAS, please run the code below:
%YOUTH_LCA_OUT(INDT=, UID=, NUMLC=3, PA= , PATXT=VIG_GIRLS, OUTDT=);
Parameters input:
INDT: (summarized) accelerometer data
UID: unique ID of target study data
NUMLC: number of latent classes assigned (Fixed, please do not change)
PA: common pre-fix of 7-day accelerometer measures from the target study data
PATXT: common character for names of latent class variable and its posterior probabilities (Fixed, please do not change)
OUTDT: Output dataset with latent class variable and its posterior probabilities generated

The following latent class variables in the table below will be generated in the dataset OUTDT accordingly.

**Variable Name**	**Description**	**Values**
**Latent classes on % Vigorous activity – Girls**		
LC_VIG_GIRLS	Latent classes based on % Vigorous activity -- girls	Categorical:<br>1 = Class 1<br>2 = Class 2<br>3 = Class 3
PP_VIG_GIRLS1	Posterior probability in latent class 1 -- based on % Vigorous activity (girls)	Continuous
PP_VIG_GIRLS2	Posterior probability in latent class 2 -- based on % Vigorous activity (girls)	Continuous
PP_VIG_GIRLS3	Posterior probability in latent class 3 -- based on % Vigorous activity (girls)	Continuous

❖ <u>Percent of vigorous activity – In school (>=4012 counts/minute) out of total wearing time</u>

Example: in SAS, please run the code below:
%YOUTH_LCA_OUT(INDT=, UID=, NUMLC=3, PA= , PATXT=VIG_INSCH, OUTDT=);
Parameters input:
INDT: (summarized) accelerometer data
UID: unique ID of target study data
NUMLC: number of latent classes assigned (Fixed, please do not change)
PA: common pre-fix of 7-day accelerometer measures from the target study data
PATXT: common character for names of latent class variable and its posterior probabilities (Fixed, please do not change)
OUTDT: Output dataset with latent class variable and its posterior probabilities generated

The following latent class variables in the table below will be generated in the dataset OUTDT accordingly.

Variable Name	Description	Values
**Latent classes on % Vigorous activity – In school**		
LC_VIG_INSCH	Latent classes based on % Vigorous activity -- in school	Categorical:<br>1 = Class 1<br>2 = Class 2<br>3 = Class 3
PP_VIG_INSCH1	Posterior probability in latent class 1 -- based on % Vigorous activity (in school)	Continuous
PP_VIG_INSCH2	Posterior probability in latent class 2 -- based on % Vigorous activity (in school)	Continuous
PP_VIG_INSCH3	Posterior probability in latent class 3 -- based on % Vigorous activity (in school)	Continuous

❖ Percent of vigorous activity – Out of school (>=4012 counts/minute) out of total wearing time

Example: in SAS, please run the code below:
%YOUTH_LCA_OUT(INDT=, UID=, NUMLC=3, PA= , PATXT=VIG_GIRLS, OUTDT=);
Parameters input:
INDT: (summarized) accelerometer data
UID: unique ID of target study data
NUMLC: number of latent classes assigned (Fixed, please do not change)
PA: common pre-fix of 7-day accelerometer measures from the target study data
PATXT: common character for names of latent class variable and its posterior probabilities (Fixed, please do not change)
OUTDT: Output dataset with latent class variable and its posterior probabilities generated

The following latent class variables in the table below will be generated in the dataset OUTDT accordingly.

Variable Name	Description	Values
**Latent classes on % Vigorous activity – Out of school**		
LC_VIG_OUTSCH	Latent classes based on % Vigorous activity -- out of school	Categorical:<br>1 = Class 1<br>2 = Class 2<br>3 = Class 3
PP_VIG_OUTSCH 1	Posterior probability in latent class 1 -- based on % Vigorous activity (out of school)	Continuous
PP_VIG_OUTSCH 2	Posterior probability in latent class 2 -- based on % Vigorous activity (out of school)	Continuous
PP_VIG_OUTSCH 3	Posterior probability in latent class 3 -- based on % Vigorous activity (out of school)	Continuous
