# Supplementary material for: Applying latent class assignments for accelerometry data to external populations: Data from the National Health and Nutrition Examination Survey 2003–2006
Source: Data Brief. 2016 Nov 9;9:926–30. doi: 10.1016/j.dib.2016.11.007 (PMC5118612; doi:10.1016/j.dib.2016.11.007)
Supplement: Supplementary file 3 — Supplementary material [file mmc2.zip › LCA_Package_Adult/Documentation_Adult_LCA_macro.pdf]

Adult Latent Classes Analysis Macro Documentation

Purpose:

This document describes the process of implementing a procedure to classify adults from an external study population into latent classes derived from national accelerometry data. These latent class assignments were derived from latent class analysis (LCA) using accelerometer data (ActiGraph AM7164) from adults >=18 years old participating in the 2003-2006 National Health and Nutrition Examination Survey (NHANES), with details explained elsewhere (Evenson KR, Wen F, Metzger JS, and Herring AH. Physical activity and sedentary behavior patterns using accelerometry from a national sample of United States adults. Int J Behav Nutr Phys Act. 2015 Feb 15;12(1):20. http://www.ijbnpa.org/content/12/1/20). The macro assigns individuals to a latent class and provides the corresponding posterior probabilities based on their pattern of accelerometry over seven consecutive days.

The latent classes, and subsequently the macros were derived for a number of different day-specific accelerometer measures including average counts per minute, percent of moderate to vigorous physical activity (MVPA) (Troiano et al., 2008; >=2020 counts/minute), percent of MVPA (Matthews et al., 2005; >=760 counts/minute), percent of sedentary behavior (Matthews et al., 2008; <100 counts/minute), and percent of sedentary bouts. Due to the more flexible LCA options, the latent classes were generated using Mplus version 7.11, but the macro was developed for use in SAS. The cutpoint values were based on the following references:

Troiano R, Berrigan D, Dodd K, Masse L, Tilert T, McDowell M: Physical activity in the United States measured by accelerometer. Med Sci Sports Exerc 2008, 40(1):181-188.

Matthews C: Calibration of accelerometer output for adults. Med Sci Sports Exerc 2005, 37(11 Suppl):S512-S522.

Matthews C, Chen K, Freedson P, Buchowski M, Beech B, Pate R, Troiano R: Amount of time spent in sedentary behaviors in the United States, 2003-2004. Am J Epidemiol 2008, 167(7):875-881.

Prerequisites to apply the macro to external study data:

- ✓ Requires that the study data are on adults age 18 years or older
- ✓ Requires that the study data contains up to 7 consecutive days of accelerometry from an ActiGraph using the vertical axis only
- ✓ Requires the accelerometer data are cleaned and summarized as follows:
    - Each participant has measures collected for 7 consecutive days, where accelerometry data are ordered by day of the week, with Sunday assigned as day 1, Monday as day 2 and so on and so forth with Saturday as day 7.
    - Adherent data is defined as a participant who wore the accelerometer for at least 3 of 7 days for at least 8 hours per day. Non-adherent data is suggested to be excluded from the analysis. Any data on non-adherent days will be required to set as missing (denoted as "." in SAS) and remain as a missing measure in the dataset.

Data Dictionary and SAS Macro Application:
Step 1: Download the example dataset and required specificity statistics data as below.

a. **Summary of Files**

Subfolder	File Name	Description
\PGM\	Generate_LCA_Macros_NHANES_Adults.sas	SAS program used to generate latent class variables
\DATA\	example_adult_cpm.sas7bdat	Example SAS dataset served as a toy dataset for users to play around with the macro
\STATS\	stats_cpm.sas7bdat	Statistics extracted from Mplus LCA model on counts/minute
	stats_mvto.sas7bdat	Statistics extracted from Mplus LCA model on %MVPA (Troiano)
	stats_mvcm.sas7bdat	Statistics extracted from Mplus LCA model on %MVPA (Matthews)
	stats_sd.sas7bdat	Statistics extracted from Mplus LCA model on %sedentary behavior
	stats_sdb.sas7bdat	Statistics extracted from Mplus LCA model on %sedentary bouts

b. **Example Dataset**
Among the 2003-06 NHANES participants, 50 adults >=18 years wore an accelerometer and were purposefully selected to be in this example dataset. The example dataset (example_adult_cpm.sas7bdat) is provided to demonstrate how the SAS macro can be implemented and used. A data dictionary for the dataset is provided below.

**Variable Name**	**Description**	**Values**
SEQN	Unique ID	Continuous
CNTMIN_1	Counts per minute on Sunday	Continuous
CNTMIN_2	Counts per minute on Monday	Continuous
CNTMIN_3	Counts per minute on Tuesday	Continuous
CNTMIN_4	Counts per minute on Wednesday	Continuous
CNTMIN_5	Counts per minute on Thursday	Continuous
CNTMIN_6	Counts per minute on Friday	Continuous
CNTMIN_7	Counts per minute on Saturday	Continuous

Step 2: Please run the macro (Generate_LCA_Macros_NHANES_Adults.sas) on a specific accelerometer measure. The macro will be able to assign the most likely latent class to each individual as well as provide their corresponding associated posterior probabilities based on the following accelerometer measures:

- Average counts per minute

  Example: in SAS, please run the code below:
  %ADULT_LCA_OUT(INDT=, UID=, NUMLC=6, PA= , PATXT=CPM, OUTDT=);
  Parameters input:
  INDT: (summarized) accelerometer data
  UID: unique ID of target study data
  NUMLC: number of latent classes assigned (Fixed, please do not change)

PA: common pre-fix of 7-day accelerometer measures from the target study data
PATXT: common character for names of latent class variable and its posterior probabilities
(Fixed, please do not change)
OUTDT: Output dataset with latent class variable and its posterior probabilities generated

The following latent class variables in the table below will be generated in the dataset OUTDT accordingly.

Variable Name	Description	Values
**Latent classes on average counts per minute (CPM)**		
LC_CPM	Latent classes on CPM	Categorical:<br>1 = Class 1<br>2 = Class 2<br>3 = Class 3<br>4 = Class 4<br>5 = Class 5<br>6 = Class 6
PP_CPM1	Posterior probability in latent class 1 – based on CPM	Continuous
PP_CPM2	Posterior probability in latent class 2 – based on CPM	Continuous
PP_CPM3	Posterior probability in latent class 3 – based on CPM	Continuous
PP_CPM4	Posterior probability in latent class 4 – based on CPM	Continuous
PP_CPM5	Posterior probability in latent class 5 – based on CPM	Continuous
PP_CPM6	Posterior probability in latent class 6 – based on CPM	Continuous

- <u>Percent of moderate-to-vigorous physical activity (MVPA; Troiano >=2020 counts/minute) out of total wearing time</u>

Example: in SAS, please run the code below:
%ADULT_LCA_OUT(INDT=, UID=, NUMLC=5, PA= , PATXT=MVTO, OUTDT=);
Parameters input:
INDT: (summarized) accelerometer data
UID: unique ID of target study data
NUMLC: number of latent classes assigned (Fixed, please do not change)
PA: common pre-fix of 7-day accelerometer measures from the target study data
PATXT: common character for names of latent class variable and its posterior probabilities
(Fixed, please do not change)
OUTDT: Output dataset with latent class variable and its posterior probabilities generated

The following latent class variables in the table below will be generated in the dataset OUTDT accordingly.

Variable Name	Description	Values
**Latent classes on % MVPA (Troiano)**		

LC_MVTO	Latent classes on % MVPA (Troiano)	Categorical:<br>1 = Class 1<br>2 = Class 2<br>3 = Class 3<br>4 = Class 4<br>5 = Class 5
PP_MVTO1	Posterior probability in latent class 1 – based on % MVPA (Troiano)	Continuous
PP_MVTO2	Posterior probability in latent class 2 -- based on % MVPA (Troiano)	Continuous
PP_MVTO3	Posterior probability in latent class 3 -- based on % MVPA (Troiano)	Continuous
PP_MVTO4	Posterior probability in latent class 4 -- based on % MVPA (Troiano)	Continuous
PP_MVTO5	Posterior probability in latent class 5 -- based on % MVPA (Troiano)	Continuous

❖ <u>Percent of moderate-to-vigorous physical activity (MVPA; Matthews >=760 counts/minute) out of total wearing time</u>

Example: in SAS, please run the code below:
%ADULT_LCA_OUT(INDT=, UID=, NUMLC=6, PA= , PATXT=MVCM, OUTDT=);
Parameters input:
INDT: (summarized) accelerometer data
UID: unique ID of target study data
NUMLC: number of latent classes assigned (Fixed, please do not change)
PA: common pre-fix of 7-day accelerometer measures from the target study data
PATXT: common character for names of latent class variable and its posterior probabilities
(Fixed, please do not change)
OUTDT: Output dataset with latent class variable and its posterior probabilities generated

The following latent class variables in the table below will be generated in the dataset OUTDT accordingly.

**Variable Name**	**Description**	**Values**
**Latent classes on % MVPA (Matthews)**		
LC_MVCM	Latent classes on % MVPA (Matthews)	Categorical:<br>1 = Class 1<br>2 = Class 2<br>3 = Class 3<br>4 = Class 4<br>5 = Class 5<br>6 = Class 6
PP_MVCM1	Posterior probability in latent class 1 -- based on % MVPA (Matthews)	Continuous
PP_MVCM2	Posterior probability in latent class 2 -- based on % MVPA (Matthews)	Continuous
PP_MVCM3	Posterior probability in latent class 3 -- based on % MVPA (Matthews)	Continuous

PP_MVCM4	Posterior probability in latent class 4 -- based on % MVPA (Matthews)	Continuous
PP_MVCM5	Posterior probability in latent class 5 -- based on % MVPA (Matthews)	Continuous
PP_MVCM6	Posterior probability in latent class 6 -- based on % MVPA (Matthews)	Continuous

- <u>Percent of sedentary behavior (Matthews <100 counts/minute) out of total wearing time</u>

  Example: in SAS, please run the code below:
  %ADULT_LCA_OUT(INDT=, UID=, NUMLC=5, PA= , PATXT=SD, OUTDT=);
  Parameters input:
  INDT: (summarized) accelerometer data
  UID: unique ID of target study data
  NUMLC: number of latent classes assigned (Fixed, please do not change)
  PA: common pre-fix of 7-day accelerometer measures from the target study data
  PATXT: common character for names of latent class variable and its posterior probabilities (Fixed, please do not change)
  OUTDT: Output dataset with latent class variable and its posterior probabilities generated

  The following latent class variables in the table below will be generated in the dataset OUTDT accordingly.

**Variable Name**	**Description**	**Values**
**Latent classes on % Sedentary behavior**		
LC_PSD	Latent classes on % Sedentary behavior	Categorical:<br>1 = Class 1<br>2 = Class 2<br>3 = Class 3<br>4 = Class 4<br>5 = Class 5
PP_PSD1	Posterior probability in latent class 1 -- based on % Sedentary	Continuous
PP_PSD2	Posterior probability in latent class 2 -- based on % Sedentary	Continuous
PP_PSD3	Posterior probability in latent class 3 -- based on % Sedentary	Continuous
PP_PSD4	Posterior probability in latent class 4 -- based on % Sedentary	Continuous
PP_PSD5	Posterior probability in latent class 5 -- based on % Sedentary	Continuous

- Percent of sedentary bouts (Matthews <100 counts/minute) out of total wearing time, where sedentary bouts is defined as >=30 minutes with at least 80% of the minutes falling below the sedentary threshold, allowing for >5 consecutive minutes above the threshold. Additionally, the bout had to start and end with sedentary behavior.

  Example: in SAS, please run the code below:
  %ADULT_LCA_OUT(INDT=, UID=, NUMLC=7, PA= , PATXT=SDB, OUTDT=);
  Parameters input:
  INDT: (summarized) accelerometer data
  UID: unique ID of target study data
  NUMLC: number of latent classes assigned (Fixed, please do not change)
  PA: common pre-fix of 7-day accelerometer measures from the target study data
  PATXT: common character for names of latent class variable and its posterior probabilities (Fixed, please do not change)
  OUTDT: Output dataset with latent class variable and its posterior probabilities generated

  The following latent class variables in the table below will be generated in the dataset OUTDT accordingly.

Variable Name	Description	Values
**Latent classes on % Sedentary behavior bouts**		
LC_SDB	Latent classes on % Sedentary bouts	Categorical:<br>1 = Class 1<br>2 = Class 2<br>3 = Class 3<br>4 = Class 4<br>5 = Class 5<br>6 = Class 6<br>7 = Class 7
PP_SDB1	Posterior probability in latent class 1 -- based on % Sedentary bouts	Continuous
PP_SDB2	Posterior probability in latent class 2 -- based on % Sedentary bouts	Continuous
PP_SDB3	Posterior probability in latent class 3 -- based on % Sedentary bouts	Continuous
PP_SDB4	Posterior probability in latent class 4 -- based on % Sedentary bouts	Continuous
PP_SDB5	Posterior probability in latent class 5 -- based on % Sedentary bouts	Continuous
PP_SDB6	Posterior probability in latent class 4 -- based on % Sedentary bouts	Continuous
PP_SDB7	Posterior probability in latent class 5 -- based on % Sedentary bouts	Continuous
